# Explainable AI-Based Hyperspectral Classification Reveals Differences in Spectral Response over Phenological Stages

**DOI:** 10.3390/biology15060454

**Published:** 2026-03-11

**Authors:** Rameez Ahsen, Pierpaolo Di Bitonto, Pierfrancesco Novielli, Michele Magarelli, Donato Romano, Martina Di Venosa, Anna Maria Stellacci, Nicola Amoroso, Alfonso Monaco, Bruno Basso, Roberto Bellotti, Sabina Tangaro

**Affiliations:** 1 Dipartimento di Scienze del Suolo, della Pianta e degli Alimenti, Università degli Studi di Bari Aldo Moro, 70125 Bari, Italy; rameez.ahsen@uniba.it (R.A.); pierpaolo.dibitonto@uniba.it (P.D.B.); pierfrancesco.novielli@uniba.it (P.N.); michele.magarelli@uniba.it (M.M.); martina.divenosa@uniba.it (M.D.V.); sabina.tangaro@uniba.it (S.T.); 2 Istituto Nazionale di Fisica Nucleare, Sezione di Bari, 70125 Bari, Italy; nicola.amoroso@uniba.it (N.A.); alfonso.monaco@uniba.it (A.M.); roberto.bellotti@uniba.it (R.B.); 3 Dipartimento di Farmacia—Scienze del Farmaco, Università degli Studi di Bari Aldo Moro, 70125 Bari, Italy; 4 Dipartimento Interateneo di Fisica “M. Merlin”, Università degli Studi di Bari Aldo Moro, 70125 Bari, Italy; 5 Department of Plant, Soil and Microbial Sciences, College of Agriculture & Natural Resources, Michigan State University, East Lansing, MI 48824, USA; basso@msu.edu

**Keywords:** machine learning, precision agriculture, feature attribution, canopy reflectance, fertilizer management, stratification strategies

## Abstract

Optimizing nitrogen (N) fertilization is essential for sustaining durum wheat yield and grain quality while reducing the environmental impacts associated with N over-application. Hyperspectral sensing provides a rapid and non-destructive approach for monitoring crop N status. However, high-dimensional data, phenology-dependent spectral responses, and spatial autocorrelation in field measurements limit robust nitrogen classification and interpretation. This study evaluated hyperspectral-based nitrogen status classification in durum wheat under Mediterranean field conditions and identified key spectral regions using explainable artificial intelligence. A field experiment was conducted in Southern Italy using ten N fertilization rates (0–180 kg N ha^−1^). Canopy reflectance was acquired at the booting and heading stages from georeferenced sampling locations. Three nitrogen stratification strategies (binary Low–High, Extreme, and three-level) were evaluated using Random Forest, SVM-RBF, and XGBoost classifiers. Model performance was assessed using spatially independent Leave-One-Plot-Out cross-validation at both the sample and plot levels, with plot-level predictions derived through majority voting. Classification robustness was strongly influenced by the stratification strategy and phenological stage. The binary Low–High stratification achieved the highest sample-level accuracy, with a maximum of 0.78 at booting (SVM-RBF) and 0.75 at heading (SVM-RBF), whereas the Extreme stratification produced intermediate performance, with maximum accuracies of 0.73 at booting (SVM-RBF) and 0.63 at heading (XGBoost). Plot-level aggregation improved performance, reaching up to 0.90 at booting and 1.00 at heading. SHAP analysis highlighted red, red-edge, and near-infrared wavelengths as the dominant contributors, with increased reliance on longer wavelengths at the heading. Overall, explainable machine learning provides a robust framework for hyperspectral nitrogen monitoring in durum wheat.

## Simple Summary

Nitrogen fertilizer has a critical role in determining grain yield and quality in cereal crops, but overfertilization can significantly impact environmental quality. This study tested whether proximal hyperspectral reflectance measurements can be used to identify wheat nitrogen status in the field. Wheat was grown with different nitrogen fertilizer rates, and measurements were taken at two growth stages, booting and heading. Three nitrogen stratification strategies (binary Low–High, Extreme, and three-level) were evaluated using Random Forest, SVM-RBF, and XGBoost classifiers. The results showed that separating nitrogen levels into only two groups, low and high, worked best and gave the most reliable results. Trying to split nitrogen levels into three groups gave poor results because the groups were too similar. Combining several measurements from the same plot improved the final decision compared with using single measurements. Overall, the study shows that simple nitrogen grouping and early measurements can support better nitrogen monitoring in wheat fields.

## 1. Introduction

In developing countries, wheat is the second most important food crop after rice, and it is widely recognized as a key contributor to the human diet, accounting for approximately 20% of daily caloric and protein intake [1]. The Food and Agricultural Organization (FAO) estimates that the demand for wheat will reach around 840 million tons by 2050. This will require developing countries to increase wheat production, primarily through improvements in yield per unit area [2]. Sufficient fertilizer inputs are required to meet this goal, focusing on nitrogen (N) due to its critical role as a macronutrient in determining grain yield and quality in cereal crops [3]. Notably, N deficiencies reduce chlorophyll, starch, and protein content, highlighting N’s role in both plant structural development and metabolic processes [4]. Therefore, N fertilization is widely required by wheat farmers, with N often being supplied in amounts that exceed real crop requirements. Although increasing N fertilization rates improves yield components, such as grain number and size [5] and grain N concentrations, N overapplication may reduce N uptake efficiency, increase the risk of groundwater pollution due to leaching [6,7], promote weed growth, delay maturity, and make the wheat more susceptible to diseases. This results in economic losses and makes agronomic management less sustainable. Hence, the early identification and the accurate measurement of crop N deficiencies or oversupplies are required to spatially and temporally optimize N fertilization [8].

Traditional methods for determining N status rely on plant tissue laboratory analysis, but while these methods produce reliable measurements, they are invasive, expensive, and time-consuming [7]. Therefore, accurately identifying actual N needs and diagnosing the spatial and temporal variability of N status can be challenging [9,10]. Additionally, due to the destructive nature of the sampling, it is not possible to repeat measurements on the same plant or at the same site, preventing the monitoring of dynamic changes in N crop status during the growing season. Spectral technologies have proven useful for rapid and non-destructive detection of crop N status, making it possible to gain insight into N management [11] and to optimize fertilization practices [12] in cropping systems. Canopy and leaf spectra are closely linked to crop N status [13], as an increase in leaf N content results in higher concentrations of chlorophyll pigments and Rubisco enzymes, leading to enhanced photosynthetic rates and changes in internal leaf structure development [14,15]. All these factors together determine vegetation reflectance properties [16,17]. Chlorophyll absorption affects the reflectance spectrum in the visible (Vis) region, while the point of maximum slope between the red chlorophyll absorption region and the region of high near-infrared (NIR) reflectance corresponds to the red-edge position (690–750 nm), which has also been found to be highly correlated with chlorophyll content [18]. On the other hand, NIR reflectance is primarily governed by leaf internal structure. A spectrum characterized by high reflectance in the NIR region and low reflectance in the VIS region is associated with N-sufficient crops [19,20], while the onset of stress conditions induces changes in the light absorption properties of the incident radiation [21], resulting in higher reflectance in Vis wavelengths and lower in NIR. Among spectral technologies, hyperspectral sensors provide significant opportunities for accurate, robust, and rapid determinations of N status in winter wheat [22,23] by capturing leaf or canopy reflectance in hundreds of narrow wavelengths and providing a continuous vegetation spectral signature. However, hyperspectral datasets are characterized by high dimensionality, strong multicollinearity, and redundant information, which can pose challenges to data analysis and result interpretation [24,25].

In recent years, the integration of advanced machine learning (ML) frameworks and explainable artificial intelligence (XAI) approaches has gained increasing adoption across the agrotech and agrifood domains, driven by the growing use of data-driven strategies for crop management and monitoring. These methodologies enable farmers and researchers to model complex relationships in high-dimensional agricultural datasets, capturing interactions among genotype, phenotype, soil characteristics, and water availability while maintaining model transparency and interpretability, which are essential for building trust and facilitating knowledge transfer in precision agriculture. Recent studies have demonstrated the effectiveness of ML and XAI for crop yield prediction [26], genotype-to-phenotype prediction and phenotype understanding for crop breeding [27,28], soil-driven modeling and environmental sensitivity analysis [29], and agricultural water management through digital twin approaches [30]. ML algorithms have also shown great potential for processing complex spectral data from various sources and detecting plant stress through handling non-linear relationships between spectral reflectance and physiological or structural crop traits [31,32]. Furthermore, optimized crop management and resource-use sustainability using data-driven ML methods have been widely reported [33], while emerging paradigms, such as federated explainable AI frameworks, are increasingly explored to enhance transparency, efficiency, and sustainability in smart agriculture systems [34].

ML approaches are now widely used in hyperspectral nitrogen estimation because they can represent complex and nonlinear relationships between high-dimensional spectral reflectance and crop nitrogen traits. Such relationships are difficult to describe using vegetation indices or parametric regression models, particularly when narrowband hyperspectral data exhibit strong multicollinearity and noise. These characteristics make ML-based methods suitable for field-scale nitrogen assessment.

ML is especially common under conditions involving heterogeneous canopy structures, variable soil backgrounds, and changing illumination. Across recent crop studies, ML is now routinely used as the modeling layer that converts hyperspectral measurements into nitrogen indicators such as plant nitrogen concentration [35], leaf nitrogen content [36], canopy nitrogen concentration [37], or nitrogen stress indices [38]. For winter wheat, Chen et al. [39] evaluated parametric regression, linear methods, SMLR, PLSR, RF, and SVM using UAV hyperspectral imagery across growth stages. They report that ML regressors often deliver higher explained variance and lower error in several stages, particularly when models are tuned per phenological window. For maize, A study [40] demonstrated a UAV hyperspectral workflow for estimating leaf and canopy nitrogen content, and explicitly tested robustness across sites, treatments, and growth stages. Work on nitrogen stress representation is also moving beyond single indices. For example, optimized nitrogen stress indicators have been derived from UAV-based hyperspectral imagery under complex field conditions, where interactions between water and nitrogen availability can confound the spectral response [41].

Most hyperspectral nitrogen studies in wheat focus on regression-based estimation of nitrogen traits, while comparatively less attention has been given to nitrogen status classification under realistic field validation. However, nitrogen-related spectral signatures vary across phenological stages due to changes in canopy structure and biomass, which can reduce class separability and limit model transferability between growth stages. In addition, classification performance is sensitive to nitrogen class stratification and may be overestimated when random sample-wise validation is applied to spatially autocorrelated field spectra. Furthermore, the limited interpretability of machine-learning models restricts understanding of the spectral regions driving nitrogen discrimination across phenological stages. Therefore, the objectives of this study were to evaluate hyperspectral nitrogen status classification in durum wheat under field conditions using spatially independent Leave-One-Plot-Out cross-validation at both sample and plot levels, and to quantify the influence of nitrogen stratification strategy and phenological stage on classification robustness. A second objective was to identify the key spectral regions contributing to nitrogen discrimination across phenological stages using SHAP-based wavelength attribution derived from out-of-fold predictions.

## 2. Materials and Methods

This section outlines the experimental design and analytical workflow used to classify crop nitrogen status from hyperspectral canopy reflectance. The approach combines a field-based nitrogen fertilization experiment under Mediterranean conditions with proximal hyperspectral measurements, multiple nitrogen stratification strategies, supervised machine-learning models, and plot-level validation. Emphasis is placed on realistic agronomic conditions, strict data independence, and wavelength-level interpretability of classification results.

### 2.1. Site Conditions Experimental Framework

The experiment was conducted over a single winter wheat growing cycle (autumn 2009–summer 2010). The crop was sown in December 2009 and harvested in July 2010 at the Cereal Research Centre in the Apulia region of Southern Italy (41°27′ N, 15°30′ E), located at an elevation of 90 m above sea level. The site represents typical Mediterranean cereal-growing conditions [42] with dry conditions prevailing in late spring and summer, while winter and autumn have wetter and milder environments. Long-term meteorological data indicate a mean annual precipitation of 531 mm, ranging from 272 to 803 mm, and a mean annual temperature of 15.8 °C, with monthly averages ranging between 8.0 and 24.4 °C.

The soil is an alluvial clay–loam classified as a chromic calcixerert [43,44]. In the upper 0.40 m of the soil profile, sand, silt, and clay contents are 238, 459, and 303 g kg^−1^, respectively. The soil contains 27.4 g kg^−1^ of organic matter and 1.1 g kg^−1^ of total nitrogen [44]. Additional soil properties include extractable phosphorus of 23.8 mg kg^−1^, a cation exchange capacity of 22.4 cmol(+) kg^−1^, calcium carbonate content of 11.4 g 100 g^−1^ soil, pH of 8.8, and low electrical conductivity of 0.2 dS m^−1^. Further details about the experimental site are reported in [44]. These characteristics are representative of intensively cultivated durum wheat soils in southern Italy.

**Figure 1:**
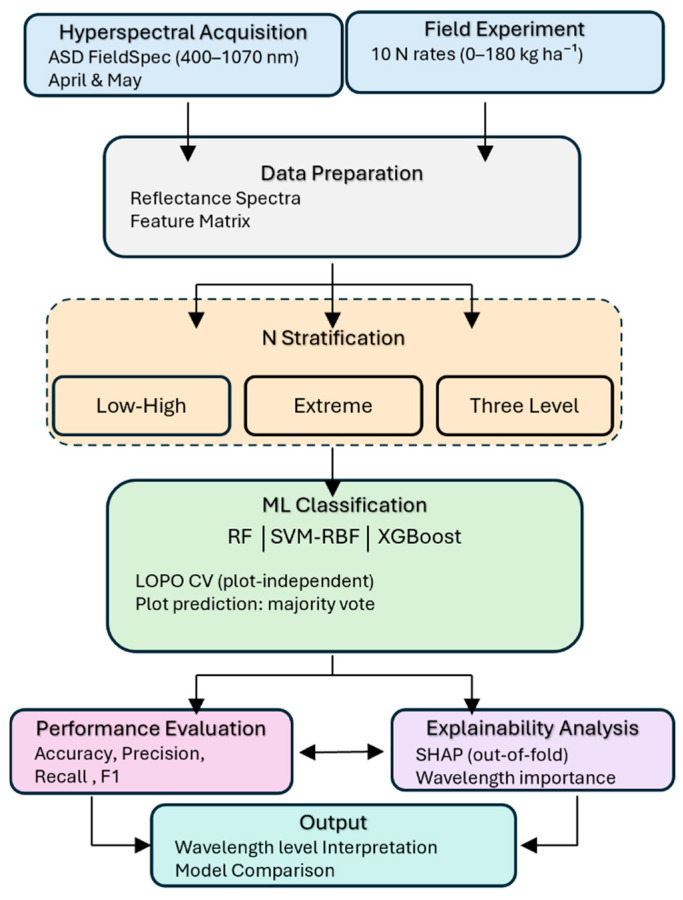
Overview of the experimental and analytical workflow used for hyperspectral-based classification of nitrogen status in durum wheat.

Durum wheat (*Triticum durum* Desf.), cultivar PR22D89, was grown under rainfed conditions following standard agronomic practices for the region. Nitrogen was applied as ammonium nitrate at ten fertilization rates: 0, 60, 80, 90, 100, 110, 120, 140, 160, and 180 kg N ha^−1^. Each nitrogen rate was assigned to a single plot measuring 10 m × 80 m, resulting in ten unreplicated treatments. Nitrogen was applied in two split doses: two-thirds of the total rate at the end of tillering and the remaining one-third at the booting stage to synchronize its availability at the crop’s critical growth stages [45]. An overview of the methodological workflow is presented in Figure 1. The Figure summarizes the main stages of the study, from field experimentation and hyperspectral canopy reflectance acquisition to nitrogen class definition and supervised model development.

### 2.2. Hyperspectral Canopy Reflectance Measurements

Hyperspectral canopy reflectance data were collected on two sampling dates, 21 April 2010 and 2 May 2010, corresponding to two key phenological stages of durum wheat development: booting (GS 40; DOY 111, 21 April) and heading (GS 55; DOY 122, 2 May). Canopy spectral measurements were acquired at 100 georeferenced sampling locations, evenly distributed across the ten nitrogen treatments, with ten sampling points per plot. Data were acquired using a portable hyperspectral radiometer (ASD FieldSpec HandHeld; ASD Inc., Boulder, CO, USA), which records reflectance in the 325–1075 nm spectral range using a 512-channel photodiode array. The instrument provides a spectral resolution of approximately 3.5 nm at 700 nm and a sampling interval of 1.5 nm. Reflectance measurements were obtained using a bare fiber-optic probe with a 25° field of view. The sensor was positioned in nadir orientation approximately 0.5 m above the canopy [44,46], corresponding to a ground sampling area of about 385 cm^2^. A calibrated barium sulfate reference panel was used to convert raw radiance measurements into reflectance values. At each sampling location, ten consecutive spectra were recorded and averaged to obtain a representative canopy reflectance signature.

Data acquisition was performed under clear, cloud-free conditions between 11:00 and 14:00 local time to minimize variability due to solar angle and illumination conditions.

Before statistical analysis, the ultraviolet (UV) portion of the spectrum (325–394 nm), generally affected by a low signal-to-noise ratio, was excluded. Conversely, the upper range of the spectrum (1005–1075 nm) was retained to further investigate the spectral behavior within the NIR region. Reflectance data in the interval 395–1074 nm were then averaged over 10 nm to reduce collinearity and overfitting. In this way, 68 derived reflectance variables were obtained, with the name indicating the central wavelength. The resulting dataset consists exclusively of the derived canopy reflectance spectra data associated with binary nitrogen input classes and was used to investigate wavelength-based discrimination of nitrogen fertilization levels. In addition, a separate data analysis was performed on the reflectance data restricted to the 395–1004 nm interval, which is generally considered noise-free. Very close results, in terms of accuracy, were obtained, and for this reason, the results are not reported in this study.

### 2.3. Nitrogen Stratification Strategies

To assess the influence of nitrogen class definition on classification performance, three nitrogen stratification strategies were evaluated.

Binary Low–High nitrogen stratification was defined using a threshold of 110 kg N ha^−1^ as an operational boundary between lower and higher nitrogen input regimes. Under Mediterranean rainfed wheat conditions, grain yield responses typically increase up to approximately 100 kg N ha^−1^, with marginal or non-significant yield gains at higher nitrogen rates [47,48,49]. Because this range represents a response transition rather than a discrete breakpoint, nitrogen treatments of 0, 60, 80, 90, 100, and 110 kg N ha^−1^ were assigned to the Low nitrogen class, while treatments of 120, 140, 160, and 180 kg N ha^−1^ were assigned to the High nitrogen class. This assignment minimizes misclassification of boundary treatments and reduces within-class spectral heterogeneity near the yield response plateau. The resulting class structure comprised six Low-nitrogen plots and four High-nitrogen plots, corresponding to approximately 60 Low-N and 40 High-N hyperspectral samples per acquisition date, which limits class imbalance during model training.

Extreme nitrogen stratification was implemented by restricting the analysis to the most contrasting nitrogen input levels, corresponding to the lower and upper tails of the nitrogen treatment gradient, with the aim of maximizing inter-class separability. Under this strategy, plots receiving 0, 60, and 80 kg N ha^−1^ were assigned to the Low nitrogen class, while plots receiving 140, 160, and 180 kg N ha^−1^ were assigned to the High nitrogen class. Intermediate nitrogen treatments were excluded. This approach follows an extreme-groups (tail-selection) design, in which classification is performed using observations from the outer portions of a gradient to increase the contrast between classes and reduce the ambiguity associated with transitional conditions, at the expense of reduced sample size [50,51].

From an agronomic and spectral perspective, extreme nitrogen deficiency or excess produces stronger changes in crop physiology and canopy structure than a moderate nitrogen supply, resulting in clearer differences in canopy reflectance magnitude and spectral shape. Remote-sensing studies have shown that canopy reflectance responds non-linearly to nitrogen availability, with spectral sensitivity being highest at low and high nitrogen levels, while intermediate levels often exhibit overlapping spectral signatures that are more difficult to distinguish [52]. As a result, restricting classification to extreme nitrogen regimes reduces spectral overlap and improves class separability in hyperspectral feature space.

The nitrogen treatments excluded from the extreme stratification, 90, 100, 110, and 120 kg N ha^−1^, were subsequently considered as representing moderate nitrogen supply conditions. These intermediate treatments occupy the central portion of the nitrogen gradient and are expected to produce canopy reflectance responses that are transitional between low and high extremes, thereby increasing within-class variability and inter-class proximity. Explicit separation of extreme and moderate nitrogen regimes allows for evaluation of the classification performance under both maximum-contrast conditions and more realistic agronomic scenarios characterized by gradual nitrogen response transitions.

### 2.4. Feature Space and Machine-Learning Models

Hyperspectral canopy reflectance data covering the 400–1070 nm spectral range were used as the sole input features for all classification models. Each variable was treated as an independent feature, resulting in a high-dimensional input space that retained the complete spectral response of the crop canopy. Reflectance spectra were used in their raw form, represented as 10 nm band-averaged reflectance variables (68 predictors, 395–1074 nm), without applying spectral normalization, filtering, smoothing, or derivative transformations. Although preprocessing techniques are frequently employed to enhance the signal-to-noise ratio and emphasize the absorption features, they introduce additional transformation parameters and methodological choices that may influence model behavior. In this study, the objective was to evaluate the intrinsic discriminative capacity of the measured reflectance signal under minimal assumptions and to maintain a direct correspondence between SHAP-derived feature importance and physically measured wavelengths. Consequently, all observed spectral variability, including within-plot and between-plot differences, was available to the learning algorithms. No feature transformation or dimensionality reduction was performed before model fitting. All models were therefore trained on the full spectral resolution, allowing subsequent model interpretation analyses to be conducted directly in wavelength space.

Three supervised machine-learning classifiers were evaluated: Random Forest (RF), Support Vector Machine with a radial basis function kernel (SVM-RBF), and Extreme Gradient Boost (XGB). These models represent complementary learning paradigms commonly applied in hyperspectral remote sensing and enable the assessment of nitrogen classification performance under different approaches to handling nonlinearity, feature interactions, and model regularization.

RF is an ensemble classifier that aggregates predictions from multiple decision trees trained on bootstrap-resampled subsets of the training data. At each node split, a randomly selected subset of spectral features is considered, which decorrelates individual trees and reduces sensitivity to highly correlated wavelength bands. This structure allows RF to exploit redundant spectral information while remaining robust to noise and multicollinearity, both of which are characteristic of hyperspectral reflectance data. Final class labels are determined by majority voting across the ensemble. RF was included for its ability to handle high-dimensional, correlated spectral features and its robustness to noise without requiring explicit feature selection.

SVM-RBF was selected for its effectiveness in nonlinear classification problems with limited numbers of independent training samples [53], a common characteristic of plot-based hyperspectral datasets. SVM-RBF with a radial basis function kernel formulates classification as a margin-maximization problem in a transformed feature space. The RBF kernel implicitly maps input spectra into a higher-dimensional space, enabling the nonlinear separation of classes that may not be linearly separable in the original wavelength domain. Model complexity is controlled by regularization and kernel parameters, which balance margin maximization against classification error. This formulation is well-suited to hyperspectral datasets, where the number of spectral features greatly exceeds the number of independent training units.

XGBoost is a tree-based boosting algorithm that constructs an ensemble of decision trees sequentially, with each tree trained to correct residual errors from previous trees [54]. The model optimizes a regularized objective function that accounts for both prediction accuracy and model complexity. Shrinkage and regularization parameters constrain tree growth and mitigate overfitting, which is particularly important when modeling high-dimensional hyperspectral data with limited independent samples. XGBoost was included as a gradient-boosting approach capable of modeling complex nonlinear feature interactions while incorporating regularization to mitigate overfitting.

### 2.5. Validation and Explainability Analysis

Model performance was evaluated using Leave-One-Plot-Out (LOPO) cross-validation, a validation strategy designed to enforce strict independence between the training and testing data at the experimental-plot level [55,56]. In each LOPO iteration, all hyperspectral spectra acquired from one plot were withheld as the test set, while the spectra from the remaining plots were used for model training and hyperparameter optimization. This procedure was repeated until each plot served once as the held-out test plot.

LOPO cross-validation was adopted to address the strong spatial and spectral autocorrelation inherent in field-based hyperspectral measurements. Spectra collected within the same plot are expected to share similar canopy structure, illumination conditions, and soil background effects. As a result, random sample-wise cross-validation would likely yield overly optimistic performance estimates by allowing information leakage between the training and testing data. In contrast, LOPO evaluates models on entirely unseen plots, providing a more realistic estimate of generalization performance across independent experimental units.

Classification performance was assessed at both the sample and plot levels using multiple complementary metrics. At the sample level, predicted and observed nitrogen classes were compared for individual hyperspectral spectra from the held-out test plot in each LOPO fold. At the plot level, sample-level predictions were aggregated using a majority voting scheme, whereby the nitrogen class most frequently predicted among all spectra from a plot was assigned as the final plot-level prediction. Overall accuracy, precision, recall, and F1 score were computed to provide a comprehensive evaluation of classification performance, particularly under potentially imbalanced class distributions. All metrics were computed within each LOPO fold and summarized using mean values and standard deviations across folds for each model, nitrogen stratification strategy, and acquisition date.

Model interpretability was investigated using SHapley Additive exPlanations (SHAP) to quantify the contribution of individual wavelengths to nitrogen classification decisions. SHAP has been shown to provide meaningful wavelength-level feature attribution in hyperspectral and remote-sensing classification tasks [57,58]. To ensure interpretability under true generalization conditions, SHAP analysis was conducted exclusively on out-of-fold test data generated within the LOPO framework. In each fold, SHAP values were computed only for spectra belonging to the held-out test plot, using the model trained on the remaining plots. This restriction avoided in-sample explainability bias and ensured that feature attributions reflected model behavior on unseen data.

SHAP analyses were performed separately for the April (booting) and May (heading) datasets to account for differences in crop phenological stage, canopy structure, and spectral response between acquisition dates. For each model and month, SHAP values were initially computed at the sample level and subsequently aggregated across LOPO folds. Global wavelength importance was derived by calculating the mean absolute SHAP value for each wavelength across all test samples and folds, capturing the overall magnitude of wavelength contributions, independent of direction. To facilitate comparison across models and acquisition dates, global SHAP importance curves were normalized to the range [0, 1] on a per-model basis, preserving relative wavelength rankings within each model. In addition to global importance profiles, SHAP beeswarm plots were generated to examine the distribution and directionality of wavelength contributions at the sample level, illustrating how variations in reflectance at specific wavelengths influenced predictions toward Low or High nitrogen classes and assessing the consistency of spectral drivers across LOPO folds.

## 3. Results

This section reports on the performance of hyperspectral-based nitrogen classification across different nitrogen stratification strategies. Detailed analyses are then presented for the binary Low–High classification, including sample- and plot-level performance under spatially independent validation. Spectral reflectance differences between nitrogen treatments are subsequently examined to support the interpretation of classification outcomes. Finally, SHAP-based analyses are used to identify globally important wavelengths and to assess the directional contribution of individual spectral regions to model predictions.

### 3.1. Classification Performance Across Nitrogen Stratification Strategies

The results are presented across different nitrogen stratification strategies and evaluation scales. Classification performance was strongly influenced by the nitrogen stratification strategy, with consistent trends observed across classifiers and acquisition dates. This effect was evident at both the sample and plot levels and was further reflected in the variability across LOPO cross-validation folds.

At the sample level, the binary Low–High nitrogen stratification yielded the highest classification accuracy across all models in both April and May (Table 1). Classification accuracies are reported as mean values with their corresponding standard deviation (SD). In April, the mean accuracies were 0.70 for RF, 0.78 for SVM-RBF, and 0.74 for XGBoost. In May, mean accuracies ranged from 0.71 for RF and 0.75 for SVM to 0.71 for XGBoost across models. Detailed class-wise performance is provided in the corresponding confusion matrices (Appendix A). Classification performance declined substantially when nitrogen was divided into three classes. Under the three-level stratification, the mean sample-level accuracies remained below 0.40 for all models and both months, indicating limited separability among the Low, Medium, and High nitrogen classes under the evaluated conditions.

The Extreme nitrogen stratification produced intermediate results. In April, sample-level accuracies were comparable to those obtained with the binary strategy, particularly for RF and SVM-RBF. However, performance decreased in May and variability across the LOPO folds increased, suggesting reduced robustness when only the lowest and highest nitrogen levels were considered, especially at later growth stages.

At the plot level, the differences among stratification strategies were more pronounced (Table 2). The binary Low–High approach again achieved the strongest performance. In April, SVM-RBF and XGBoost correctly classified 9 out of 10 plots, while RF correctly classified 7 plots. In May, SVM-RBF achieved perfect classification of 10/10 plots, whereas RF and XGBoost each misclassified 2 plots.

Plot-level performance under the Three-level stratification was consistently low. Depending on the classifier and acquisition date, only two to five plots were correctly classified, highlighting substantial confusion among nitrogen classes. The Extreme stratification yielded intermediate plot-level results. While XGBoost achieved relatively higher accuracy under this configuration, the reduced number of plots led to increased sensitivity to individual misclassifications (Appendix A).

**Figure 2:**
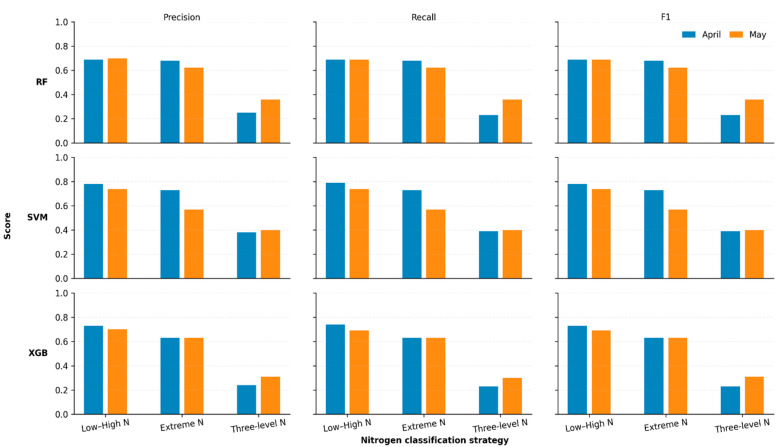
Sample-level macro-averaged precision, recall, and F1 score for hyperspectral nitrogen status classification under three nitrogen stratification strategies (Low–High N, Extreme N, and Three-level N). Metrics are displayed by column, with precision on the left, recall in the center, and F1 score on the right. Results are shown separately for two acquisition datasets to enable comparison of performance consistency across acquisition conditions. Rows correspond to the evaluated classifiers, with RF in the top row, SVM-RBF in the middle row, and XGB in the bottom row.

Sample-level macro-averaged Precision, Recall, and F1-scores for all classifiers and stratification strategies are shown in Figure 2. Macro averaging was used to ensure equal contribution from each class, regardless of class imbalance. Across both acquisition dates, the Low–High stratification consistently produced the highest macro Precision, Recall, and F1 values for all models, with scores generally exceeding 0.65. The Extreme strategy resulted in lower macro-averaged metrics, while the Three-level strategy exhibited the weakest performance, with the macro F1 scores typically below 0.40. Among the evaluated classifiers, SVM-RBF consistently achieved the highest macro-averaged scores, particularly in April. A modest decline in macro-Precision, Recall, and F1 was observed from April to May across most strategies.

### 3.2. Sample- and Plot-Level Performance Under Binary Low–High Nitrogen Classification

At the sample level, SVM-RBF achieved the highest mean accuracy in April, with a value of 0.78 ± 0.19, followed by XGBoost at 0.74 ± 0.13 and RF at 0.70 ± 0.18. In May, sample-level accuracies were more similar across classifiers, ranging from 0.71 to 0.75, and were accompanied by higher standard deviations for all models, particularly for RF and SVM-RBF, approximately 0.21, indicating increased variability across LOPO folds.

At the plot level, classification performance improved following majority voting. In April, SVM-RBF and XGBoost correctly classified 9 of the 10 plots, whereas RF correctly classified 7 plots. In May, SVM-RBF correctly classified all 10 plots, while RF and XGBoost each correctly classified 8 plots. Despite the increased variability associated with the binary nature of LOPO evaluation, the plot-level performance was consistently equal to or higher than the corresponding sample-level performance across classifiers and acquisition dates.

### 3.3. Spectral Characteristics of Low and High Nitrogen Treatments

**Figure 3:**
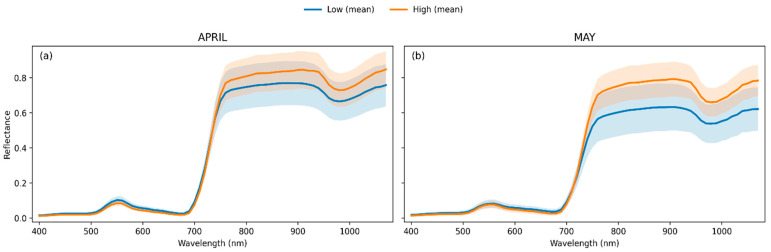
Mean spectral reflectance profiles for Low and High nitrogen treatments across the hyperspectral range for (**a**) the booting stage (April) and (**b**) the heading stage (May) acquisitions. Shaded regions represent variability around the mean reflectance.

Mean hyperspectral reflectance spectra for the Low and High nitrogen treatments are shown in Figure 3 for the booting (April) and heading (May) stages over the 400–1000 nm range, with shaded areas indicating the standard deviation around the mean reflectance for each class. In the visible region (400–700 nm), both nitrogen classes exhibited low reflectance values at both phenological stages, with a local maximum in the green region around 550 nm (Figure 3a,b). In April, the mean reflectance in the visible range was slightly higher for the Low nitrogen class across most wavelengths, although differences between classes were small and largely within the standard deviation envelopes. In May, visible-region reflectance patterns were similar, with closely overlapping mean spectra and variability ranges.

More pronounced separation between nitrogen classes was observed in the red-edge region (approximately 700–750 nm). In April at the booting stage, the transition from red to near-infrared reflectance occurred at shorter wavelengths for the High nitrogen class, resulting in higher mean reflectance values immediately beyond the red edge. This separation extended into the near-infrared region (750–900 nm), where the High nitrogen class consistently exhibited higher reflectance than the Low nitrogen class, with moderate within-class variability.

In May, at the heading stage, near-infrared reflectance values were lower for both nitrogen classes compared with April. Although separation between Low and High nitrogen treatments in the red-edge and near-infrared regions remained observable, differences in mean reflectance were reduced, and greater overlap between the standard deviation envelopes was evident.

Across both acquisition dates, differences between nitrogen classes were minimal in the visible region and became more pronounced beyond approximately 700 nm. The wavelength range between roughly 730 and 900 nm showed the most consistent separation in mean reflectance between Low and High nitrogen treatments.

### 3.4. Global Wavelength Importance Derived from SHAP Analysis

**Figure 4:**
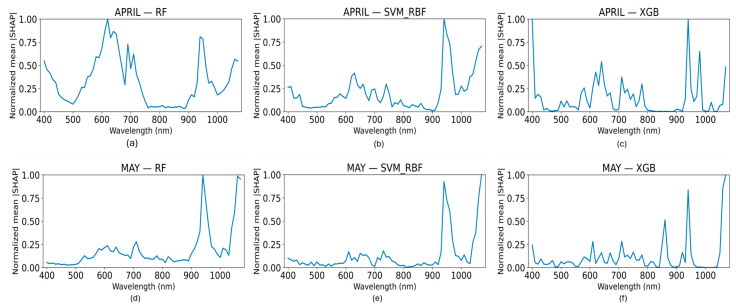
Global wavelength importance profiles derived from SHAP analysis for hyperspectral nitrogen classification. Each curve represents the normalized mean absolute SHAP value across wavelengths, indicating the relative contribution of individual spectral bands to model predictions. (**a**) RF for the April dataset; (**b**) SVM-RBF for the April dataset; (**c**) XGB for the April dataset; (**d**) RF for the May dataset; (**e**) SVM-RBF for the May dataset; (**f**) XGB for the May dataset. SHAP importance values were computed on out-of-fold test samples within the LOPO framework and aggregated across folds to obtain global wavelength rankings. SHAP analysis was performed for the Low–High nitrogen stratification, which served as the primary classification configuration.

Global wavelength importance was assessed using SHAP values computed from out-of-fold predictions under the binary Low–High nitrogen classification. For each classifier and acquisition date, SHAP values were aggregated across all samples and expressed as normalized mean absolute values to enable comparison across wavelengths, which can be seen in Figure 4.

In April, at the booting stage, RF showed the highest importance in the red region (610–650 nm) and a second strong peak in the NIR region (940–960 nm), indicating nitrogen sensitivity mainly through chlorophyll absorption and N-related canopy effects. A secondary contribution was observed for the red-edge region, within the 660–710 nm interval, and the blue wavelengths. SVM was dominated by the NIR peak (940–950 nm), showing that nitrogen discrimination relied mostly on this region. Although of lower relevance, two peaks were observed in the visible region, for the red (610–650 nm) and blue (400 nm) wavelengths. XGB highlighted strong importance in the blue region (400 nm) and again in the NIR, with moderate contribution from the red region (600–650 nm). Overall, all three models consistently identified the NIR as the most important nitrogen-sensitive region, while RF emphasized the red band, and XGB additionally captured nitrogen signals in the blue band.

In May, at the heading stage, RF showed dominant importance in the NIR (940–960 nm) and the long NIR (1050–1060 nm), with only minor contributions from the red and red-edge region. SVM was strongly concentrated in the NIR and long NIR, indicating that nitrogen prediction at the heading stage is mainly driven by these two wavelength regions. XGB also emphasized the long NIR and NIR, while additionally highlighting a noticeable contribution around 860–880 nm. Overall, all three models consistently identified the NIR (940–960 nm) and long NIR (1050–1060 nm) as the most important nitrogen-sensitive regions at the heading stage, with SVM showing the most focused dependence on these bands and XGB capturing additional nitrogen-related information across a broader NIR range. Despite differences in the smoothness and distribution of importance profiles among the models, overlapping wavelength intervals with elevated SHAP values were observed.

### 3.5. Directional Wavelength Contributions Revealed by SHAP Beeswarm Analysis

**Figure 5:**
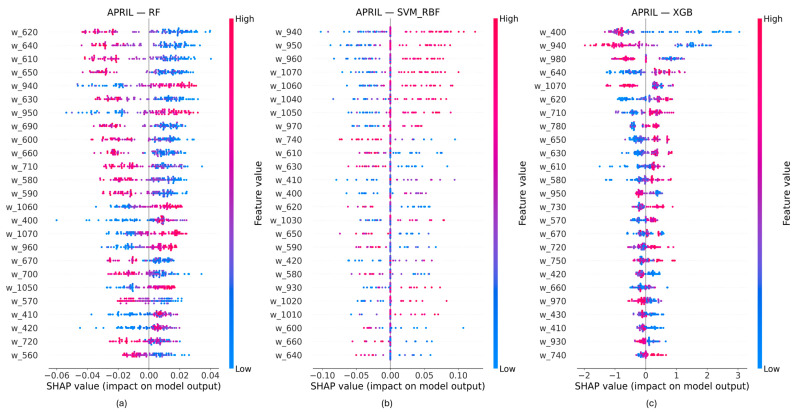
Directional wavelength contributions derived from SHAP beeswarm analysis for the acquisition at the booting stage (April 2010). Each point represents the SHAP value of a wavelength feature for individual samples, with color indicating the relative feature value. (**a**) RF model; (**b**) SVM-RBF model; (**c**) XGB model.

**Figure 6:**
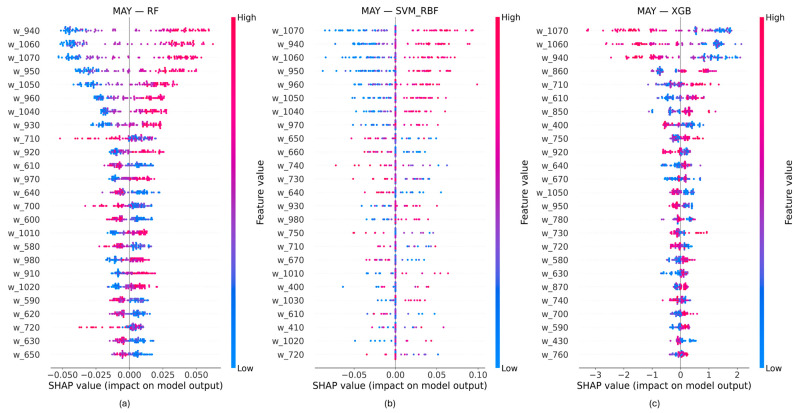
Directional wavelength contributions derived from SHAP beeswarm analysis for the acquisition at the heading stage (May 2010). Each point represents the SHAP value of a wavelength feature for individual samples, with color indicating the relative feature value. (**a**) RF model; (**b**) SVM-RBF model; (**c**) XGB model.

Directional wavelength contributions were examined using SHAP beeswarm plots derived from out-of-fold predictions under the binary Low–High nitrogen classification (Figure 5 and Figure 6). For each model and acquisition date, SHAP values are shown by wavelength, with the color indicating the feature value and horizontal position representing the direction and magnitude of each contribution.

At the booting stage (April), clear directional differences were observed between spectral regions. For the RF model, wavelengths in the visible range (approximately 560–700 nm) were predominantly associated with negative SHAP values, with higher reflectance corresponding to more negative contributions. In contrast, near-infrared wavelengths (approximately 940–960 and 1050–1070 nm) exhibited predominantly positive SHAP values, with higher reflectance associated with larger positive contributions. The RF model showed broad dispersion in SHAP values across samples. For SVM-RBF, directional contributions were concentrated mainly in the near-infrared region, while visible and red-edge wavelengths exhibited SHAP values clustered near zero. XGBoost displayed high-magnitude SHAP values at a limited number of wavelengths, with strong directional separation, while most wavelengths showed near-zero contributions.

At the heading stage (May), directional SHAP patterns shifted toward longer wavelengths for all models. Visible and red-edge wavelengths exhibited low SHAP magnitudes with limited directional separation, whereas near-infrared wavelengths showed clearer separation between positive and negative contributions. Compared with booting (April), SHAP value dispersion across samples was generally reduced, particularly for wavelengths below approximately 800 nm. The significant contribution of the NIR wavelengths in the interval 940–960 nm and 1050–1070 nm at this phenological stage was highlighted by the three models.

Across both acquisition dates, visible wavelengths consistently showed low directional contributions, while near-infrared wavelengths dominated SHAP-based importance. Differences among models were evident in the number of wavelengths exhibiting non-zero SHAP values and in the spread of SHAP contributions across samples.

## 4. Discussion

This study investigated the nitrogen status classification in wheat using hyperspectral reflectance data collected at different growth stages. The results show that classification performance depends strongly on how nitrogen classes are defined, when data are acquired, and how predictions are aggregated, highlighting the need to align methodological choices with the intended application of nitrogen monitoring.

Binary Low–High nitrogen classification consistently produced the most stable and reliable results across models and acquisition dates. This indicates that coarse nitrogen grouping leads to more separable spectral patterns under field conditions, particularly when spatial independence is enforced through plot-wise validation. Similar findings have been reported in recent hyperspectral studies, where broader nitrogen groupings reduced class overlap and improved robustness [59]. Meanwhile, the poor performance of the three-level stratification reflects substantial spectral overlap among adjacent nitrogen levels, consistent with the continuous nature of crop nitrogen responses [60].

The Extreme nitrogen strategy showed intermediate performance, particularly early in the season. Although restricting the analysis to the lowest and highest nitrogen levels increased agronomic contrast, this configuration reduced the number of independent plots and altered the variance structure within each class. The removal of intermediate nitrogen rates decreased the density of samples near the decision boundary and increased within-class heterogeneity among the remaining treatments. As a result, model estimation became more sensitive to plot-level variability, which may explain the lower stability and reduced accuracy observed for the Extreme configuration despite the larger nominal nitrogen contrast. This sensitivity reflects reduced sample availability and increasing canopy heterogeneity, a limitation also noted in recent stress-classification [4] studies using hyperspectral data.

Differences between the booting (April) and heading (May) stages emphasize the interaction between crop development stage and nitrogen stratification strategy. Seasonal effects are not uniform across configurations. While the Low–High stratification shows stable or slightly improved performance in May, the Extreme stratification exhibits reduced accuracy for RF and SVM, and the Three-level configuration shows moderate improvement. These findings indicate that seasonal effects are contingent upon class definition and classifier characteristics rather than uniform across configurations. A more detailed inspection of the SHAP-derived wavelength importance revealed a seasonal redistribution in the RF model. At booting (April), dominant contributions were observed in the 600–660 nm region, particularly near 620 nm, indicating a strong influence of red absorption features. At heading (May), the importance shifted toward longer near-infrared wavelengths (approximately 940–960 nm and 1050–1060 nm), while visible-region contributions decreased. Mean spectral profiles show that nitrogen separation at heading (May) is primarily expressed across the near-infrared plateau, consistent with this shift in model reliance from pigment-dominated to canopy structural reflectance characteristics.

The longer near-infrared region (approximately 940–1070 nm) is primarily influenced by internal leaf scattering and canopy structural properties rather than chlorophyll absorption. Reflectance in this domain depends on mesophyll structure, leaf thickness, and canopy density, which affect multiple scattering of radiation within the foliage. As the crop progresses toward later growth stages, increased biomass and canopy closure enhance structural contributions to near-infrared reflectance.

Wavelengths near 970 nm are also associated with weak water absorption features, suggesting that leaf water content may contribute to variability in this region. Although water status was not directly measured, its interaction with canopy structure may partially explain the increased importance of longer NIR wavelengths at heading (May). Overall, the seasonal shift in SHAP importance reflects the increasing influence of structural and canopy-level reflectance effects as the crop develops.

In a previous study performed on the same data applying correlation analysis, principal component analysis (PCA) and stepwise discriminant analysis (SDA), the spectral bands and intervals which characterized the response of winter wheat treated with increasing N levels were: red (650, 680 nm), red edge (710–730 nm), and NIR (770, 820, 940 nm), with a lower contribution of green (550 nm) and yellow (610 nm) intervals, at booting (April); while green (510, 520 nm), yellow (580, 600 nm), and NIR (820, 890 nm) at heading (May) [44]. These results highlight the shift from visible and red edge towards NIR regions of the spectrum with the advancement of the crop growing cycle and confirm that differences in growth stages can cause a significant change in the waveband selection. Similar results were observed by applying the MAXR algorithm, considering the response variable leaf N concentration. At both the booting (April) and heading (May) stages, the red and far-red (670–680 nm), red-edge (740, 750 nm) and NIR (900 and 960 nm) bands were selected, with the greatest significance for the red-edge (740, 750 nm) and NIR (960 nm) bands [44] and the inclusion of the initial part of the moisture-sensitive trough portion of NIR [61].

Feng et al. (2023) [62], using hyperspectral information from a field spectroradiometer, observed the potential for identifying wheat N stress, even when simultaneous yellow mosaic disease stresses occurred. They demonstrated that the 725–1000 nm range is sensitive to N deficiency and that the SVM model with pre-processing techniques for separating the soil contribution allows for discrimination between different N stresses with higher OA (96.97%) than KNN and RF.

Aggregating predictions at the plot level consistently improved performance relative to sample-level classification. Majority voting reduced the influence of local spectral noise and within-plot variability, supporting the use of plot-level decision units in operational nitrogen monitoring. Recent geospatial machine-learning studies [63] likewise emphasize the importance of spatially independent validation and aggregation to avoid optimistic accuracy estimates and ensure model transferability. Spectral analysis indicated minimal separation between nitrogen classes in the visible region, with clearer differences emerging beyond approximately 700 nm. The red-edge and near-infrared regions consistently showed greater discriminative power, in agreement with previous hyperspectral studies linking these regions to nitrogen-related canopy properties [64].

SHAP analysis further supported these observations by identifying near-infrared wavelengths as the dominant contributors to model predictions. Directional SHAP results showed that higher near-infrared reflectance generally increased the likelihood of High nitrogen classification. Red and red-edge regions were also relevant at the booting stage, particularly in the RF model, while visible wavelengths contributed to a lesser extent at the heading stage. Although attribution patterns varied across models, they converged on similar spectral regions, indicating largely model-independent relevance. The study [58] applying explainable ML to hyperspectral data reports comparable concentration of feature importance in these wavelength ranges.

In this experimental design, each nitrogen rate was applied to a single plot without treatment-level replication. Consequently, nitrogen effects cannot be fully disentangled from potential plot-specific influences such as soil heterogeneity or localized environmental variability. Although LOPO validation ensures spatial independence in model evaluation, it does not substitute for true agronomic replication. The results should therefore be interpreted as treatment-level discrimination under the specific field conditions of this study.

The relatively small number of independent experimental units (ten plots) should also be considered when comparing classifiers. While spatial independence was maintained during validation, the limited number of plots may influence the statistical stability of model ranking and the robustness of conclusions regarding the best-performing classifier. Accordingly, comparisons among RF, SVM, and XGB are most appropriately interpreted within the scope of this dataset. In addition, the binary threshold of 110 kg N ha^−1^ was defined based on agronomic considerations within the experimental context. Although this threshold represents a meaningful management boundary under the studied conditions, its transferability to other environments, soil types, or climatic regions was not evaluated. Broader applicability would require validation across independent sites and growing seasons.

Overall, the findings indicate that reliable nitrogen classification from hyperspectral data benefits from coarse class definitions, early-season acquisition, and spatial aggregation. Increasing class granularity or relying on late-season imagery substantially reduces robustness. These results provide practical guidance for designing hyperspectral nitrogen-monitoring workflows under realistic field conditions.

## 5. Conclusions

Hyperspectral-based nitrogen status classification in durum wheat was assessed under field conditions, with particular emphasis on the influence of a nitrogen stratification strategy and the spectral drivers of model predictions. Model performance was evaluated using spatially independent LOPO cross-validation at both the sample and plot levels, and wavelength-level interpretability was investigated using a SHAP analysis applied exclusively to out-of-fold test data.

The results demonstrate that nitrogen class definition is a primary determinant of classification robustness. Across classifiers and acquisition datasets, the binary Low–High stratification consistently achieved the highest and most stable performance, whereas the three-level stratification showed substantial performance degradation, indicating limited separability between adjacent nitrogen classes under realistic field variability. The extreme-class stratification produced intermediate performance and exhibited increased variability across LOPO folds, suggesting sensitivity to plot-level heterogeneity and phenological differences.

Acquisition timing and crop phenological stage further affected classification outcomes. Performance was generally stronger in the early acquisition, while later-season classification became more variable and less discriminative, consistent with reduced spectral separation in the red-edge and near-infrared regions as the canopy structure became more complex. Plot-level aggregation through majority voting improved classification relative to the sample-level predictions, supporting the use of spatial aggregation to reduce local noise and within-plot variability in operational monitoring.

SHAP-based wavelength attribution provided consistent evidence that nitrogen discrimination was primarily driven by the red, red-edge, and near-infrared regions. Global and directional SHAP patterns highlighted these wavelengths as dominant contributors to model predictions, confirming their relevance for nitrogen-related classification in durum wheat. Overall, the study indicates that a reliable hyperspectral nitrogen status classification under field conditions is favored by coarse nitrogen stratification, early-season acquisition, and plot-level aggregation, and that explainable modeling can support the identification of informative spectral regions for nitrogen monitoring workflows.

## Figures and Tables

**Table 1:** Sample-level, model-wise mean classification accuracy and standard deviation under different nitrogen stratification strategies for April and May acquisitions.

N Stratification	Dataset	RF ± SD	SVM ± SD	XGBoost ± SD
Low–High N	April	0.70 ± 0.18	0.78 ± 0.19	0.74 ± 0.13
	May	0.71 ± 0.21	0.75 ± 0.21	0.71 ± 0.17
Extreme N	April	0.68 ± 0.25	0.73 ± 0.27	0.63 ± 0.20
	May	0.62 ± 0.26	0.57 ± 0.36	0.63 ± 0.23
Three-level N	April	0.23 ± 0.22	0.38 ± 0.26	0.23 ± 0.20
	May	0.36 ± 0.22	0.40 ± 0.25	0.31 ± 0.20

**Table 2:** Plot-level, model-wise mean classification accuracy and standard deviation (SD) under different nitrogen stratification strategies for acquisitions at the booting (April) and heading (May) stages.

N Stratification	Dataset	RF ± SD	RF-Correct Plots (n/N)	SVM ± SD	SVM-Correct Plots (n/N)	XGBoost ± SD	XGBoost-Correct Plots (n/N)
Low–High N	April	0.70 ± 0.49	7/10	0.90 ± 0.32	9/10	0.90 ± 0.32	9/10
	May	0.80 ± 0.42	8/10	1.00 ± 0.00	10/10	0.80 ± 0.42	8/10
Extreme N	April	0.68 ± 0.52	4/6	0.68 ± 0.52	4/6	0.83 ± 0.41	5/6
	May	0.50 ± 0.59	3/6	0.50 ± 0.55	3/6	0.83 ± 0.41	5/6
Three-level N	April	0.20 ± 0.42	2/10	0.40 ± 0.52	4/10	0.20 ± 0.42	2/10
	May	0.50 ± 0.53	5/10	0.40 ± 0.52	4/10	0.40 ± 0.52	4/10

## Data Availability

The data used in this study will be available upon request.

## Appendix A

This appendix provides detailed confusion matrices corresponding to the classification results reported in the main manuscript. Confusion matrices are presented at both the sample level and the plot level for all nitrogen stratification strategies (Low–High, Extreme, and Three-Level) and for each classifier (RF, SVM-RBF, and XGBoost).

### Appendix A.1. Sample Level Confusion Matrix

Sample-level matrices summarize the number of individual spectra correctly and incorrectly classified within the held-out test plots. These results reflect model performance at the spectral measurement level and provide insight into class-wise prediction behavior and error distribution.

**Table A1:** Sample-level confusion matrices for the Low–High nitrogen stratification. Values represent the number of individual spectra correctly and incorrectly classified, where all spectra from one plot were excluded from model training and used exclusively for testing. Accuracy corresponds to the overall proportion of correctly classified spectra (n = 100 per month).

Month	Model	H→H	H→L	L→H	L→L	Accuracy
April	RF	25	15	15	45	0.70
April	SVM-RBF	33	7	15	45	0.78
April	XGB	30	10	16	44	0.74
May	RF	23	17	12	48	0.71
May	SVM-RBF	27	13	12	48	0.75
May	XGB	23	17	12	48	0.71

H = high nitrogen class; L = low nitrogen class. The arrow (→) indicates the predicted class.

**Table A2:** Sample-level confusion matrices for the Extreme nitrogen stratification (bottom-three vs. top-three nitrogen levels). Values represent the number of individual spectra correctly and incorrectly classified, where spectra from each test plot were excluded from model training. Accuracy corresponds to the overall proportion of correctly classified spectra (n = 60 per month).

Month	Model	H→H	H→L	L→H	L→L	Accuracy
April	RF	20	10	9	21	0.68
April	SVM-RBF	23	7	9	21	0.73
April	XGB	19	11	11	19	0.63
May	RF	17	13	10	20	0.62
May	SVM-RBF	17	13	13	17	0.57
May	XGB	18	12	10	20	0.63

H = high nitrogen class; L = low nitrogen class. The arrow (→) indicates the predicted class.

**Table A3:** Sample-level confusion matrices for the Three-Level nitrogen stratification. Values represent the number of individual spectra correctly and incorrectly classified when spectra from each test plot were excluded from model training. Accuracy corresponds to the overall proportion of correctly classified spectra (n = 100 per month).

Month	Model	H→H	H→L	H→M	L→H	L→L	L→M	M→H	M→L	M→M	Accuracy
April	RF	5	1	24	4	8	18	16	14	10	0.23
April	SVM-RBF	16	2	12	4	12	14	15	15	10	0.38
April	XGB	5	3	22	2	7	21	15	14	11	0.23
May	RF	11	5	14	5	9	16	10	14	16	0.36
May	SVM-RBF	13	4	13	5	11	14	12	12	16	0.40
May	XGB	9	8	13	4	7	19	11	14	15	0.31

H = high nitrogen class; L = low nitrogen class; M = medium nitrogen class. The arrow (→) indicates the predicted class.

### Appendix A.2. Plot-Level Confusion Matrix

Plot-level matrices are derived from majority voting across spectra within each plot. In this case, a plot is assigned to the class receiving the highest number of spectral predictions. Plot-level results, therefore, represent spatially aggregated decisions and correspond to the evaluation framework used for reporting plot-level accuracy in the main text.

**Table A4:** Plot-level confusion matrices for the Low–High nitrogen stratification obtained using Leave-One-Plot-Out (LOPO) validation. Values represent the number of correctly and incorrectly classified plots. Accuracy is reported as the number of correctly classified plots over the total number of plots (n = 10).

Month	Model	H→H	H→L	L→H	L→L	Accuracy
April	RF	3	1	2	4	7/10
April	SVM-RBF	4	0	1	5	9/10
April	XGB	4	0	1	5	9/10
May	RF	3	1	1	5	8/10
May	SVM-RBF	4	0	0	6	10/10
May	XGB	3	1	1	5	8/10

H = high nitrogen class; L = low nitrogen class. The arrow (→) indicates the predicted class.

**Table A5:** Plot-level confusion matrices for the Extreme N stratification obtained using Leave-One-Plot-Out (LOPO) validation. Values represent the number of correctly and incorrectly classified plots. Accuracy is reported as the number of correctly classified plots over the total number of plots (n = 6).

Month	Model	H→H	H→L	L→H	L→L	Accuracy
April	RF	2	1	1	2	4/6
April	SVM-RBF	2	1	1	2	4/6
April	XGB	3	0	1	2	5/6
May	RF	1	2	1	2	3/6
May	SVM-RBF	2	1	2	1	3/6
May	XGB	3	0	1	2	5/6

H = high nitrogen class; L = low nitrogen class. The arrow (→) indicates the predicted class.

**Table A6:** Plot-level confusion matrices for the Three-Level N stratification obtained using Leave-One-Plot-Out (LOPO) validation. Values represent the number of correctly and incorrectly classified plots. Accuracy is reported as the number of correctly classified plots over the total number of plots (n = 10).

Month	Model	H→H	H→L	H→M	L→H	L→L	L→M	M→H	M→L	M→M	Accuracy
April	RF	0	0	3	0	1	2	2	1	1	2/10
April	SVM-RBF	3	0	0	0	1	2	3	1	0	4/10
April	XGB	0	0	3	0	1	2	2	1	1	2/10
May	RF	2	0	1	0	1	2	1	1	2	5/10
May	SVM-RBF	2	0	1	1	1	1	2	1	1	4/10
May	XGB	2	0	1	0	0	3	1	1	2	4/10

H = high nitrogen class; L = low nitrogen class; M = medium nitrogen class. The arrow (→) indicates the predicted class.
